# Evidence-based strategies for delivering grant writing skills to clinical and translational science faculty in the Mountain West

**DOI:** 10.1017/cts.2025.10198

**Published:** 2025-11-12

**Authors:** Ruben K. Dagda, Larissa Myaskovsky, Akshay Sood, Kathrene Conway, Joseph Guerrero Lopez, Mark Burge, Lorraine S. Evangelista, Francisco S. Sy

**Affiliations:** 1 Associate Professor, Reno Howard Medical Sciences (MHS), University of Nevada, Reno, NV, USA; 2 UNM School of Medicine, Department of Internal Medicine, University of New Mexico, Albuquerque, NM, USA; 3 School of Public and Community Health Sciences, University of Montana, Missoula, MT, USA; 4 School of Public Health, University of Nevada, Las Vegas, NV, USA; 5 Department of Medicine, Endocrinology & Metabolism, University of New Mexico, Albuquerque, NM, USA; 6 Sue & Bill Gross School of Nursing, University of California Irvine, Irvine, CA, USA; 7 Department of Environmental and Occupational Health, University of Nevada Las Vegas (UNLV), Las Vegas, NV, USA

**Keywords:** Clinical and translational research, mentoring, grant writing workshop, early-stage and mid-career investigators, health disparities

## Abstract

Acquiring the skills to obtain extramural funding is a major challenge for early- and mid-career investigators. The Professional Development Core (PDC) of the NIH-funded Mountain West Clinical and Translational Research Infrastructure Network (MW CTR-IN) aims to support early-stage and mid-career investigators pursuing independent careers in clinical and translational science research. Since 2018, the PDC’s Grant Writing Workshops (GWWs) have provided CTR investigators with didactic content and interactive feedback on their NIH grant applications, helping them reach this key career milestone. Four one-day GWWs were offered in person, and two half-day GWWs were offered virtually across two days during the COVID-19 pandemic. Evaluation data for each cohort revealed that participants’ knowledge and confidence in the relevant sections of NIH R-series grants consistently improved following GWW attendance and resulted in notable enhancements in participants’ feelings of positivity toward grant writing, regardless of delivery mode (virtual vs. in-person). Follow-up data showed that 12 GWW participants acquired external funding with a 21% success rate and $12,584,938 in total funding. This manuscript provides a roadmap for planning and implementing a successful virtual or in-person GWW that positively impacts the careers of early-stage and mid-career investigators.

## Introduction

The Mountain West Clinical and Translational Research Infrastructure Network (MW CTR-IN) is a collaborative network of thirteen institutional partners from seven states in the Mountain West region. Funded by the National Institute of Health (NIH) Institutional Development Award (IDeA) program since 2013, the MW CTR-IN supports the career development of faculty engaged in clinical and translational research (CTR) and led by the University of Nevada, Las Vegas [1]. The Mountain West’s rural areas are medically underserved due to inadequate clinical infrastructure and low health equity, particularly among minorities and rural populations [2,3].

A lack of adequate training, mentoring, and a nurturing environment in CTR; insufficient time to write grant applications; lack of accessible resources; reduced networking opportunities; and insufficient team science training can hinder early-stage and mid-career investigators (ESMCIs) from achieving successful careers in CTR at research-intensive academic institutions [4–6]. Hence, to support ESMCIs in meeting their professional development needs, the network features a Professional Development Core (PDC). [1]. Guided by the Significant Learning Experiences theory to maximize the impact of learning and feedback between learners and mentors [1,7], the PDC assists ESMCIs in addressing professional development needs by connecting faculty with highly competent mentors, facilitating real-time mentoring, and providing access to educational resources, which are critical “ingredients” that can foster the success of junior and mid-career faculty to attain a successful career in CTR [5,8–11]. Acquisition of extramural funding is one of the most significant milestones that ESMCIs must achieve to secure tenure and promotion. At the same time, ESMCIs must successfully sustain scholarly productivity and secure long-term institutional support, which is necessary to grow a competitive CTR team [12,13]. Therefore, acquiring extramural funding for ESMCIs is a top priority among academic institutions in building a CTR infrastructure to reduce health disparities.

Within academic institutions, the Offices of Research and Enterprise, with support from NIH-funded programs and in coordination with the Office of Faculty Affairs, typically coordinate and organize GWWs to provide grant writing coaching and mentoring to ESMCIs. The formats of GWWs can vary and are designed to cater to specific audiences, research foci, delivery modes, funding opportunity announcements, sponsors, and the level of grant writing experience of faculty members. Traditional single-day GWWs, organized by institutions through their faculty development offices, provide didactic content to support early-career investigators in grant writing. However, these workshops are designed to accommodate a large audience (>50 faculty) and lack opportunities for participant interaction, writing time, exercises, or workshop sessions. Although one-day, didactic-rich workshops can be beneficial for developing specific skills, they may not adequately address the needs of all ESMCIs, as they may not effectively provide support, such as one-on-one mentoring and writing activities [8,9,14].

Unlike these static one-day workshops, the GWW, supported by the MW CTR-IN, provides participants with writing exercises, follow-up consultations, and one-on-one guidance. Current evidence suggests that GWWs supported by the NIH are more successful because they have greater access to funding and resources, allowing them to develop a more comprehensive curriculum that includes dynamic activities to assist participants with their grant writing applications [13,15]. Thus, the PDC offered a quasi-annual curriculum for GWWs to support ESMCIs in Western IDeA states in writing competitive NIH grants. This curriculum aimed to provide them with the necessary tools, mentorship, confidence, and knowledge to secure grant funding. Based on the NIH version of the Grant Application Writer’s Workbook [16], the GWWs aim was to provide a comprehensive guide for ESMCIs who intend to submit an NIH R-series grant in the next two funding cycles. The MW CTR-IN recruited qualified speakers and CTR mentors to provide participants with career development resources, knowledge, and training. Although the MW CTR-IN’s GWW shares common features with other NIH-supported programs (e.g., COBRE, INBRE), such as interactive coaching and an engaging curriculum, several elements make our model unique, including (a) an immersive curriculum that integrates didactic content with hands-on, interactive activities and flexible delivery formats (virtual, in-person, or hybrid); (b) on-demand one-on-one mentoring before, during, and after the workshop; and (c) access to a wide array of resources (NIH workbooks, sample grant sections, and virtual tools). Thus, this educational research paper presents data collected from six cohorts of ESMCIs who attended MW CTR-IN GWWs (in person or virtually), focusing on assessing confidence, knowledge acquisition, and their relationship to the acquisition of extramural funding from the NIH.

## Methods

### Program overview

Since 2018, the Mountain West Clinical and Translational Infrastructure Network Professional Development Core (MW CTR-IN PDC) has offered quasi-annual GWWs in person (or via Zoom during COVID-19 pandemic restrictions) at the network’s annual conference. Invited learners had to (a) be faculty ESMCIs who worked at least 0.50 FTE in one of the MW CTR-IN partner institutions; (b) have obtained or are currently working on an active CTR pilot project; and (c) committed to attend most activities during the GWW, demonstrated either in their written personal statement in their GWW application or by acknowledging their commitment in the application checklist. Participants who did not meet these criteria (e.g., administrative staff from partner schools’ departments, graduate students, or post-docs) were invited to audit the GWW but were not required to complete pre- and post-evaluations or participate in interactive sessions. GWWs consisted of either beginner workshops targeted to ESCMIs with no NIH grant application experience or advanced virtual GWWs tailored for ESMCIs who had significant preliminary data, sought support with a grant proposal’s research approach, or requested coaching on resubmitting a revised application and wanted to develop expertise in applying for new or revised grants. Following the GWW, participants had the option to seek additional support on their grant applications by getting one-on-one mentoring or consultation with PDC staff, or by submitting their new or revised application to a PDC-sponsored external review system (Advanced to Funding) [1].

### Needs assessment survey

**Figure 1.**
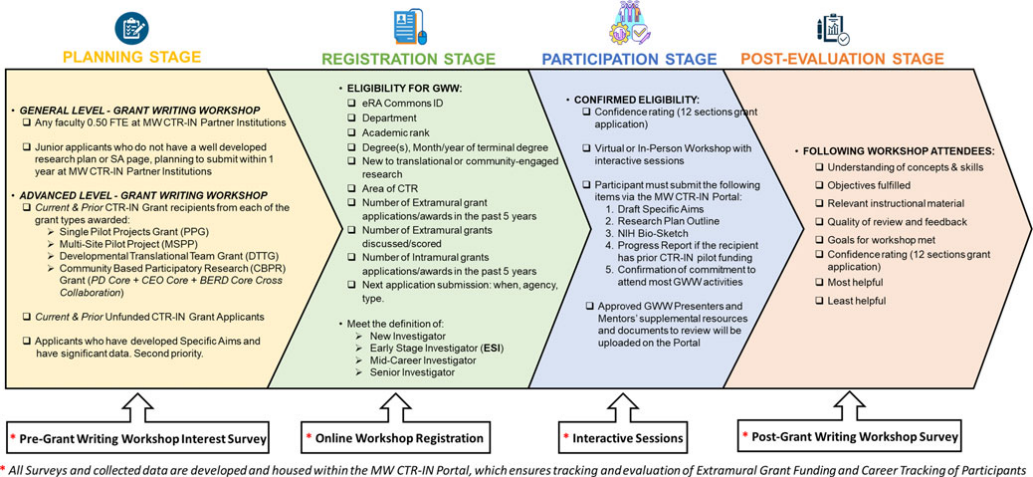
Grant writing workshop (GWW) workflow. Phases of the workflow in planning and deploying a grant writing workshop, which includes administering a pre-GWW interest survey (a.k.a. needs assessment survey) to gauge the interest and confidence of CTR faculty on various GWW topics to create tailored training content (needs assessment survey), registering for the GWW, delivering the GWW (participation), collecting and analyzing evaluation data after the GWW (post-evaluation). Details regarding the items included in the interest survey, eligibility, registration, and pre/post-evaluation process are provided for each phase of the workflow.

The GWW’s program agenda was tailored to the desired topics and grant processes of potential participants based on responses to a needs assessment survey (Figure 1, Step 1). The survey, administered through Qualtrics, was comprised of 26–30 questions sent to ESMCIs involved in CTR via email or a monthly newsletter. Participants were asked to rank a list of 20 topics in order of preference for which they needed assistance. Data on potential participants’ demographics, career stage, expertise, rank, and grant writing objectives were also collected. We deployed surveys six months before the GWW date, with several reminders, and used the results of these surveys to develop a detailed meeting agenda. We used electronic formats, including a monthly newsletter, email blasts from institutional concierges, and communication through each institution’s Office of Research and Enterprise.

### Program preparation

The MW CTR-IN GWWs were delivered either in person (one 7-hour day) or via Zoom (two half-days, 3-4 hours each). Each GWW required up to six months of extensive preparation, which entailed drafting and administering a Needs Assessment survey to interested CTR PIs, reserving the venue for in-person GWWs, recruiting speakers, and developing the agenda (see Figure 1 for a detailed description of the GWW model and logistical workflow). The PDC organized the agenda and invited GWW speakers to present on topics tailored according to the Needs Assessment surveys. Speakers were vetted based on their subject matter expertise, experience acquiring NIH funding, and mentoring proficiency.

Speakers consisted of PDC faculty, other cores of the MW CTR-IN, or outside experts for a specialized topics (per Needs Assessment requests). Topics included addressing rigor and reproducibility in grant applications, engaging the community in research, communicating with program officials, dealing with grant rejection, how to revise a reviewed grant application, and finding the most suitable study section for your application. The GWW date was announced when the Needs Assessment survey was deployed.

### Registration process

**Figure 2.**
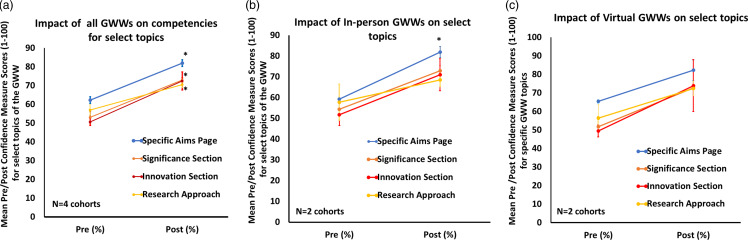
Mean pre–post confidence measure scores for key topics of the grant writing workshops. Mean pre–post confidence measure scores for key topics were analyzed from all cohorts of GWWs, virtual GWWs, or in-person GWWs (*: *p* value <0.05 for post–pre comparison, student’s *t*-test).

The registration process required applicants to submit demographic information (Figure 2) and complete a form that asked participants to evaluate their confidence in various grant writing skills and knowledge of the grant process using a 0–100 scale (0 = Cannot do at all; 100 = Highly certain can do). Admission to the GWW also required participants to upload their Specific Aims page, NIH Biosketch, and other documents depending on the type of GWW offered. Registration and document uploading were completed electronically through the MW CTR-IN Portal or Qualtrics.

### Organization of the GWW

The one-day GWW featured tailored morning talks on five to six topics, followed by two interactive sessions in the afternoons. The interactive sessions, each lasting 45 minutes, featured writing exercises and mentor consultations to help participants improve their grant application sections. Most often, the importance of writing a strong Specific Aims page, highlighting the strengths of the proposed research project, and organizing the research plan for a funding application was requested. These sessions enabled facilitators to provide input, which inspired peers and mentors to offer constructive feedback, thereby enhancing self-efficacy and improving writing [15].

Because published studies demonstrated that GWWs that offer individual consultations and interactive sessions to faculty tend to be associated with the most positive and long-term outcomes [13–15], this approach was implemented in the GWWs offered by the MW CTR-IN. Thus, each participant was assigned up to two mentors by the PDC. These mentors were assigned based on their content expertise related to the GWW participants’ area of research, as described on their SA page, to assist with their writing sessions. Additionally, two participants reciprocally reviewed another participant’s specific aims and other materials. Hence, each participant received up to three rounds of valuable written and oral comments. Each in-person GWW took place in a pre-conference the day before the annual MW CTR-IN summit to maximize participation. Speakers, participants, and administrative staff gathered in a large conference room.

Due to COVID-19 pandemic restrictions, in-person meetings were replaced by virtual meetings via Zoom (in 2021 and 2022). Virtual GWWs were offered in two half-day seminars (instead of one seven-hour session) to reduce Zoom fatigue and thereby increase participation. Four lecturers delivered 30- to 40-minute lectures on the first day. On the second day, Zoom “breakout rooms” were led by a senior faculty member who assessed each participant’s goals and facilitated meetings like “Workshopping the Specific Aims.” See Supplemental Document 1 for representative agendas for in-person and virtual GWWs.

### Post-GWW evaluations

After each in-person or virtual workshop, participants were asked to complete a post-GWW evaluation survey in order to access all electronic didactic and interactive content, including virtual materials and additional electronic grant writing tools, covered in the GWW through secure links to a Google Drive or a secure portal, which could only be accessed after completing the survey. The ‘post-GWW evaluation phase’ was the final step in the program, as shown in Figure 1. In 2021, pre- and post-evaluations were introduced to enhance the assessment of the GWWs’ impact, account for influential variables, and gather more comprehensive data on the effectiveness of the GWW. All participants completed the evaluation survey, which provided valuable information for both summative and formative purposes, supporting continuous improvement. The evaluation assessed participants’ satisfaction with the GWW’s ability to meet their goals on a Likert Scale (5 = Strongly agree, 1 = Strongly disagree). It also evaluated the impact of each topic and interactive session on participants’ confidence in learning, applying knowledge, and receiving oral feedback. Participants rated their agreement with each item on a 0–100 scale (0 = Cannot do at all; 100 = Highly certain can do).

Finally, to track the impact of GWWs on participants’ career development, the PDC monitored participants’ acquisition of NIH funding from 2021 to 2024. NIH eReporter and Grantome.com databases were used to track the type of award mechanism, project information, and the total dollar amount of awards funded.

### Analysis plan

Continuous variables (e.g., satisfaction or confidence scores) are reported using means and standard deviations for each GWW topic covered. Pairwise Student’s t-tests were used to compare the pre- and post-GWW data. Data were compared between in-person and virtual GWWs using unpaired t-tests. One-way ANOVA was used to evaluate performance scores and identify cohort differences. P-values less than 0.05 were considered significant.

## Results

The MW CTR-IN offered six GWWs to 78 faculty between 2018 and 2024. Four of the six GWWs were held in person, with the remaining presented virtually (2021-2022). The average size of in-person cohorts was 13, whereas the virtual cohorts averaged 7 participants. The lower participation observed in virtual GWWs can be attributed to the challenging circumstances of the COVID-19 pandemic, which hindered academic activity. Most (60.3%) of GWW participants were assistant professors, 23.1% were associate professors, and the remaining group (16%) consisted of full professors, other research faculty and post-docs, or administrative staff who audited the program (Table 1). Unlike the in-person GWWs, the virtual GWWs were comprised of predominantly assistant professors (80%), with the remainder classified as associate professors (Tables 1–2).

**Table 1.** Background information for grant writing workshop attendees (2018–2024)

Year	2018			2019			2021			2022			2022			2024		
GWW DATES / LOCATION / WORKSHOP LEVEL	January 18 (*In-Person*)			November 1-2 (*In-Person*)			June 28-29 (*Virtual*) ^***1***^ ***Advanced Level***			April 13-14 (*Virtual*) ^2^ ***Beginner Level***			November 16 (*In-Person*) ^***1***^ ***Advanced Level***			January 29 (*In-Person*) ^***2***^ ***Beginner Level***		
	n (%)	***CLINICIAN***	***NON- CLINICIAN***	***n* (%)**	***CLINICIAN***	***NON- CLINICIAN***	***n* (%)**	***CLINICIAN***	***NON- CLINICIAN***	***n* (%)**	***CLINICIAN***	***NON- CLINICIAN***	***n* (%)**	***CLINICIAN***	***NON- CLINICIAN***	n (%)	***CLINICIAN***	***NON- CLINICIAN***
**Total participants**	**14** *(100%)*	**6** (*43%*)	**8** (*57%*)	**12** *(100%)*	**2** (*17%*)	**10** (*83%*)	**6** *(100%)*	**2** (*33%*)	**4** (*67%*)	**9** *(100%)*	**5** (*56%)*	**4** (*44%*)	**18** (*100%*)	**11** (*61%*)	**7** (*39%*)	**19** (*100%*)	**4** (*21%*)	**15** (*79%*)
**ASSSISTANT PROFESSOR**	***8*** *(57%)*	4	4	***9*** *(75%)*	2	7	***3*** *(50%)*	0	3	***9*** *(100%)*	5	4	***11*** (*61%*)	6	5	***7*** (*36.8%*)	1	6
**ASSOCIATE PROFESSOR**	***4*** *(28%)*	1	3	***2*** *(17%)*	0	2	***3*** *(50%)*	2	1	***0*** *(0%)*	0	0	***3*** (*17%*)	2	1	***6*** (*31.6%*)	2	4
**FULL PROFESSOR**	***1*** *(7.14%)*	1	0	***0*** *(0%)*	0	0	***0*** *(0%)*	0	0	***0*** *(0%)*	0	0	***0*** (*0%*)	0	0	***2*** (*10.5%*)	1	1
**OTHER**	***1*** (7.14%)	0	1	***1*** *(8%)*	0	1	***0*** *(0%)*	0	0	***0*** *(0%)*	0	0	***4*** (*22%*)	3	1	***4*** (*21.1%*)	0	4
**# OF PARTNER INSTITUTES**	8			*6*			5			8			3			10		
**# OF TOPICS COVERED**	5			*6*			9			9			6			7		
**# OF INTERACTIVE SESSIONS**	1			*2*			2			2			2			1		
**AVG GWW SATISFACTION SCORE (** *1-5, 5 IS BEST* **)**	4.4			4.75			4.83			4.53			4.4			4.37		
**% OF POSITIVE COMMENTS**	*50%*			*100%*			*72%*			*60%*			*65%*			*61%*		
**Extramural Grant Funding (EMGF) RECEIVED TO DATE**	$3,462,745			$477,345			$183,320			*$183,817*			*$0*			*$53,426*		
**DEMOGRAPHICS OF ALL GWW PARTICIPANTS**	**ASSISTANT PROFESSOR**			**ASSOCIATE PROFESSOR**						**FULL PROFESSOR**						**OTHER**		
**ALL GWW (%)**	*60.26%*			*23.08%*						*3.85%*						*12.82%*		
**IN-PERSON GWW (%)**	*55.56%*			*23.81%*						*4.76%*						*15.87%*		
**VIRTUAL GWWS (%)**	*80%*			*20%*						*0%*						*0%*		
				***TOTAL ADVANCED LEVEL PARTICIPANTS (n = 24)***						***TOTAL BEGINNER LEVEL PARTICIPANTS (n = 28)***						***AVG MEAN SATISFACTION SCORES***		
**VIRTUAL WORKSHOPS [4 in total]**	***TOTAL CLINICIAN n (*** *%* ***)***			**7** (46%)	*2 (40%)*					*5 (60%)*						4.48 ± 0.18		
	***TOTAL NON-CLINICIAN n (*** *%* ***)***			**8** (53.34%)	*4 (50%)*					*4 (50%)*								
*IN-PERSON WORKSHOPS [2 in total]	***TOTAL CLINICIAN n (*** *%* ***)***			**15** *(40%)*	*11 (73%)*					*4 (27%)*						4.68 ± 0.21		
	***TOTAL NON-CLINICIAN n (*** *%* ***)***			**22** *(60%)*	*7 (32%)*					*15 (68%)*								

^***1*, *2***^ *Indicates grant writing experience: (* ***Advanced*** *= <2-3 Years or more /* ***Beginner*** = less than 1 year >) *Excludes data from 2018 and 2019.

EMFG: Extramural Funded Grants; GWW: Grant Writing Workshop.

**Table 2.** Background and outcomes information for GWWs participants across project years

Year	2018	2019	2020	2021	2022	2022	2023	2024
**GWW dates / location / workshop level**	January 18 (in-person)	November 1–2 (in-person)	COVID-19 pandemic	June 28–29 (virtual) *advanced level*	April 13–14 (virtual) *beginner level*	November 16 (in-person) *advanced level*	Grant re-submission	January 29 (in-person) *beginner level*
**Participants (n)**	**14**	**12**		**6**	**9**	**18**		**19**
**Extramural grant funding (EMGF) received to date**	$3,462,745	$477,345		$183,320	*$183,817*	*$0*		*$53,426*
**No. of PIs funded with grants**	3	3		1	2	1		2
**No. of grants funded**	3	3		1	2	1		2
**Success rate (PIs funded / No. of cohort members or grant submissions)**	25%	27%		20%	20%	6%		13%
**Research topics of interest among GWW participants**	PC: 0 HD: 20% Non-HD: 80% Comm: 0 Other: 0	PC: 0 HD: 43% Non-HD: 58% Comm: 0 Other: 0		PC: 0 HD: 33% Non-HD:33% Comm:16.6% Other: 16.6%	PC: 0 HD: 20% Non-HD: 80% Comm: 0 Other: 0	PC: 10 HD: 40% Non-HD: 40% Comm: 0 Other: 0		PC: 7.69 HD: 38.46% Non-HD: 30.76% Comm: 15.38% Other: 7.69%
**Research topics funded for all cohort**	1 Health Disparities, 2 Other clinical	2 Community Engaged Research, 1 Other		1 Other	2 Other	1 Other		1 Preclinical, 1 Other

Comm: Community engaged research, EMFG: Extramural Funded Grants PC: Preclinical, COVID19: Coronavirus Disease 2019, GWW: Grant Writing Workshop, HD: Health Disparities, Non-HD: Non-health Disparities, PI: Principal Investigator.

*Note:* Non-health disparities research: other clinical translational research that is not directly centered on addressing health disparities between different population groups.

In-person cohorts before 2020 attracted ESMCIs from 6–8 of the 13 partner institutions and included clinicians and non-clinicians. Clinicians comprised 17 to 60% of each GWW cohort (Table 1). In contrast, the 2024 in-person cohort exhibited greater site diversity, featuring representation from 10 partner institutions that included PIs conducting research in all areas of CTR, including community-engaged research and preclinical research (Table 1). The data presented in Table 1 demonstrate the success of the GWWs, regardless of their mode of delivery (virtual vs. in-person), as evidenced by the level of positive feedback and overall mean satisfaction score pertaining to the GWW’s program and content. The overall mean satisfaction score per cohort participants ranged from 4.37 to 4.83 on the Likert scale, with the 2021 cohort achieving the highest score. Data showed that virtual GWWs were associated with a nonsignificant mean satisfaction score compared to in-person GWWs (mean overall score 4.68 vs. 4.48, *p* = 0.228, unpaired *t*-test). The evaluation metrics included determining the proportion of positive written feedback from participants, which aligned with the quantitative data. The feedback for all cohorts was positive, ranging from 50 to 100% (Table 1). Representative positive and negative feedback from in-person and virtual GWWs is shown for two participants for each mode of GWW delivery (Supplemental Document 2). Notably, no major challenges were attributed to any participant group, and there was wide participation among clinicians and non-clinicians across ranks (Table 1).

We observed that the number of interactive sessions offered for each GWW cohort organized by the MW CTR-IN were positively associated with their mean satisfaction scores. The GWWs that offered one interactive session involving both written and oral feedback regarding participants’ grant application components yielded a mean satisfaction score of 4.4. In contrast, GWW cohorts offering multiple interactive sessions were associated with satisfaction scores ranging from 4.4 to 4.83. However this difference was non-significant in unpaired t-tests (*p* = 0.1780).

Research topics of interest among GWW participants included health disparities research (20–40%), other CTR (30–80%) and preclinical research (0–10%) (Table 2). Tracking the outcomes of 57 former participants who submitted extramural applications from 2021–2024 showed that 12 ESMCIs achieved $12,584,938 in external funding (Table 2) with a 21% success rate and $1,048,744 per funded PI. CTR themes that were funded ranged from health disparities research (∼8%), community-engaged research (17%), preclinical research (∼8%), and other CTR topics (∼67%) (Table 2). The 2018 and 2019 cohorts had the highest funding success rates, at 25 and 27%, respectively, compared to other cohorts that were delivered virtually or in person (Table 2).

Pre- and post-data assessing the impact and effectiveness of GWWs on the competencies of specific topics discussed within the same cohort of GWWs are presented in Tables 2 and across four cohorts of GWWs (Figure 2). Table 3 presents the data for all topics presented at the GWWs. For the highly ranked topics, the evaluation data showed an increase in the average confidence level of participants after each workshop for various topics, such as ‘Preparing a Specific Aims Page,’ ‘Preparing a Competitive Significance Section,’ ‘Preparing a Competitive Innovation Section,’ and ‘Preparing a Solid Research Approach Section’ (Table 3). The participants in the GWW program demonstrated the greatest increase in confidence in their ability to prepare a specific aims page, with an improvement ranging from 19 to 41%. However, a lower range of confidence improvement was observed in the ‘Preparing a Competitive Research Approach Section,’ ranging from 2 to 40%.

**Table 3.** Pre–Post mean confidence scores for grant writing workshops (2021-2024)

Year	2021		2022		2022		2024	
**Date / location / workshop level**	June 28–29 (virtual) advanced lev*el*		April 13–14 (virtual) b*eginner level*		November 16 (in-person) adva*nced level*		January 29 (in-person) beginner l*evel*	
**GWW workshop topic**	Pre	Post	Pre	Post	Pre	Post	Pre	Post
Preparing a specific aims page	66	78.33	64.86	86.22	59.22	83.78	59.21	80
How to prepare a layry							62.26	71.38
Preparing a competitive significance Section	46.5	70	57.14	75.67	58.11	71.22	50.74	74.76
Preparing a competitive innovation section	45.67	74.5	53.43	73.44	58	68.78	45.63	73.24
Preparing a solid research approach section	58.67	82.33	54.14	62.56	61.56	63	54	74
Engaging your community in research					44.44	63.89		
Utilizing new methods and instruments used in health disparities research	70.83	79.17	62.86	66.11				
Tailoring and preparing figures for a grant application	55.17	69.83	34.71	67.78			55.26	70.48
Preparing an NIH Bio-sketch	51	75.33	68.29	86.67			67.84	81.57
Preparing a budget and budget justification	60.83	68.17	43.71	56.22				
Targeting the most appropriate study section(s) for yount	41.17	78.17	41.71	66.56	56.44	75.22		
Talking to and approaching your program official					52.11	76.44		
Understanding the grant review process in study sections	49.17	78.17	50.14	76.67			50.21	72.05
Understanding the requirements of the FOA by NIH (Including Rigor)							51.95	73.33
Understanding how to deal with grant rejections	46.17	71	43.86	60.89				
Conducting appropriate biostatistical analysis of your preliminary data and/or developing an appropriate analysis plan for your application (power analysis, Bayesian and non-Bayesian methods, analysis of intra-and inter- population variance, pre-/post- tests in CTR grant applications)	57.17	72.67	45.43	57	54.11	75.22		

The percent change in mean confidence for each GWW topic is indicated in the third column for each cohort (*: *p* < 0.05 vs. pre-evaluation, Student’s *t*-test). GWW: Grant Writing Workshop, FOA: Funding Opportunity Announcement; NIH: National Institutes of health; CTR: Clinical Translational Research.

Regarding miscellaneous topics provided in different GWW sessions, pre- and post-data indicated varying degrees of improvement in confidence. The topics covered in the workshop included understanding the funding announcement requirements by the NIH, which was associated with a 22% increase in participants’ mean confidence. Other topics focused on specialized areas of health disparities research (e.g., utilization of new methods and instruments) and were associated with a 4 to 9% increase in participants’ mean confidence after completing the GWW (Table 3).

During the pandemic, our data showed that the number of virtual participants was notably lower than that of in-person participants. Furthermore, some participants in the virtual GWWs expressed concerns about the insufficient time allocated for grant writing exercises and the lack of mentor feedback. Figure 2 depicts the average gain in confidence measures when the data were grouped by session format: 1) all GWWs, 2) in-person GWWs, or 3) virtual GWWs. In brief, the aggregate data showed a notable increase in confidence among all participants for “Preparing a Specific Aims Page,” “Preparing a Significance Section,” and “Developing a Competitive Innovation Section” following the GWW (*p:* < 0.05, paired *t*-test). In contrast, differences in confidence for the “Preparing a Research Approach Section” (Figure 2a) were minimal. However, when the data were grouped by session format, a significant increase in mean confidence was observed in the topic of “Preparing Specific Aims Page” for in-person GWWs (*p:* <0.05, paired *t*-test; *N* = 4 cohorts), whereas only positive trends were observed for the same topics offered by virtual GWWs (Figure 2b–c; *N* = 2).

## Discussion

Between 2018 and 2024, 78 MW CTR-IN ESMCIs participated in a Grant Writing Workshop. Evaluation data consistently showed a positive impact on participants’ grant writing skills regardless of whether the sessions were conducted virtually or in person. Participants reported substantial gains in confidence, particularly in areas critical to NIH R-series proposals. Notably, the improvement in developing the Specific Aims page was higher than for other components, a result likely attributable to the dedicated “Workshopping the Specific Aims Page” session provided for every cohort. The data suggest that GWWs with increased opportunities for interaction were associated with greater learner satisfaction. In contrast, topics delivered without an interactive component did not yield comparable confidence gains (Table 3). These findings underscore the importance of providing personalized, real-time feedback during the grant application process. This strategy has enhanced participant preparedness, as demonstrated by both immediate satisfaction surveys and long-term funding outcomes.

Workshop participants secured extramural funding totaling $12,584,938, averaging $1,048,744 per principal investigator and achieving an overall funding success rate of 22%. The portfolio of awards included highly competitive mechanisms such as NIH R01s and an R16, as well as multiple R03s and R21s. These success metrics are comparable to those reported by other IDeA-funded mentoring programs [13], where approximately 27% of ESMCIs secured NIH funding. This parity underscores the value of an integrated approach that combines rigorous didactic instruction with interactive, mentor-supported sessions. Given that most NIH R-type grants are typically funded within three years of the initial submission [5,13,15], the external funding acquired per cohort is anticipated to increase for another three years following the conclusion of the final cohort (e.g., through 2027 for the 2024 cohort).

The structural design of the workshops significantly contributed to these outcomes. Virtual GWWs typically featured small cohorts of 6 to 9 participants and a favorable 2:1 mentor-to-mentee ratio. This configuration allowed each participant to receive individualized guidance from two mentors during interactive exercises during the GWW and in subsequent follow-up consultations. Although the virtual format inherently limits spontaneous interactions compared to in-person sessions, the structured approach ensures focused learning and consistent engagement.

Following the COVID-19 pandemic, a noticeable shift in participant preferences emerged, with increased demand for in-person sessions. In response, the PDC adopted a dual-delivery model in 2022, offering both virtual and in-person workshops to accommodate diverse preferences while maintaining instructional quality. This hybrid approach successfully leveraged the strengths of each format. Virtual sessions promoted scalability and personalized mentoring, while in-person sessions fostered richer and more spontaneous interactions. From a resource-allocation perspective, virtual workshops offer a scalable and cost-effective option, particularly when funding is limited or when participants are geographically dispersed. A hybrid delivery model that incorporates both approaches further enhances the learning experience by accommodating additional speakers and expanding one-on-one mentoring opportunities.

Importantly, continuous improvement has been central to the evolution of the GWWs. Post-workshop surveys provided actionable feedback, with participants suggesting that reducing the volume of didactic content and shortening speaker segments would allow more time for active grant-writing exercises. Additional recommendations included incorporating more breaks to improve focus and comfort (Supplemental Document 2). Informed by these insights, the most recent iterations of the workshops (2022–2024) were redesigned, resulting in increased enrollment, elevated confidence levels, especially in crafting Specific Aims, and broader faculty representation. Notably, the percentage of clinicians that participated significantly increased from 17% in earlier cohorts to as high as 61% in November of 2022.

Although some participants expressed a specific interest in pursuing grants focused on health disparities, the workshops were intentionally designed to benefit a broad spectrum of CTR principal investigators working across various phases of translational science, including community-engaged and preclinical research. Data indicate that both virtual and in-person sessions effectively address the diverse professional development needs of ESMCIs (Tables 2–3, Figure 2).

The GWWs offered by the MW CTR-IN differ in important ways from other NIH-funded programs such as COBRE and INBRE, as well as from institution-based initiatives/programs and published grant-writing models [13,15,17–19]. Although some institutions deliver full-day, standalone GWWs with didactic content presented by a single speaker to accommodate large audiences [20], most contemporary approaches involve centralized coaching over extended periods, typically 16 to 20 months, pairing participants with mentors, “super-mentors,” and biostatisticians, and incorporating formats such as dyadic mentoring, speed mentoring, online or remote facilitation, and group-based sessions. These programs generally recruit mentors from a single institution and frequently operate as standalone workshops that offer “centralized” resources with a range of dynamic or follow-up activities. In contrast, the MW CTR-IN GWWs maintain the advantages of leveraging resources (speakers, mentors, funding) across multiple institutions while maintaining small cohorts but are uniquely tailored to the specific needs, challenges, and CTR-related interests of each group, as identified through a pre-workshop needs assessment. This customization ensures that the content addresses the most relevant topics for each cohort. Although the GWWs themselves are short in duration, they are embedded within a continuity model in which beginner workshops focus on developing NIH proposal drafts and advanced workshops support revision and refinement. Mentoring is continuous before, during, and after the GWW workshops, an approach that is based on the Fink model [1], through an electronic ticketing portal that allows participants to receive real-time feedback from mentors. The program also provides a clear a path toward funding by offering participants the opportunity to submit revised proposals to the MW CTR-IN Professional Development Core for further external review through the Advance to Funding program. Although there is some overlap with other models, the MW CTR-IN’s integration of personalized agendas, dynamic activities, dyadic mentoring, and broad resource accessibility create a distinctive and comprehensive approach. Participation is further incentivized by prioritizing early-stage investigators with active or previously funded pilot projects. Through its combination of tailored content, interactive engagement, and sustained mentorship, the MW CTR-IN GWW model addresses critical CTS core competencies in research proposal development, data analysis, scientific communication, problem formulation, innovation, teamwork, and community engagement.

## Limitations

One limitation of the GWWs offered by the MW CTR-IN is their short-term programming provided to participants which aimed at providing a wealth of didactic content and mentoring in a short amount of time. In contrast, research has shown that compared to one and two-day workshops, longer programs (>3 months) with multiple mentors and guided virtual consultations improve grant success and scholarly productivity [13,15,19,21]. Despite MW CTRIN GWW being designed as one- and two-day sessions due to resource constraints, their high-impact outcomes have significantly advanced the career trajectories of ESMCIs and strengthened the overall capacity for clinical and translational research across MW institutions.

Another limitation to our study was the way we collected evaluation data from our GWWs. Summative and formative aspects of the program were evaluated as a whole and not by the speaker’s performance. Furthermore, the evaluation data were collected in an anonymous manner; therefore, it was not possible to segregate the data based on faculty demographics to identify differences in confidence in learning based on faculty rank and other demographic characteristics. Furthermore, the absence of a proper control group (e.g., participants who did not receive any programming or who received only online didactic content) limits our ability to make causal inferences. Finally, pre/post data only were collected after 2021, thereby limiting the amount of data to be analyzed to gauge participant improvement.

## Conclusion

To support ESMCIs in achieving the critical milestone of securing extramural funding, the MW CTR-IN Professional Development Core successfully delivered six GWWs since 2018. These workshops provided essential didactic training in grant writing, intensive mentorship, and hands-on coaching specifically tailored to CTR faculty across MW institutions. Overall, the integration of rigorous didactic instruction with interactive, mentor-supported activities has proven essential in equipping investigators with the skills necessary to secure extramural funding. Given similar outcomes in virtual and in-person formats, offering extended sessions should leverage virtual mentoring to maximize successful long-term outcomes for GWW participants. In order to help meet this goal, our program is able to share relevant additional training documents (e.g. PWT lectures, activities, examples of successful grants in health disparities and other) beyond this publication upon request if other investigators are interested to further implement our model.

## Supporting information

10.1017/cts.2025.10198.sm001 Dagda et al. supplementary material 1 Dagda et al. supplementary material

10.1017/cts.2025.10198.sm002 Dagda et al. supplementary material 2 Dagda et al. supplementary material

## Supplementary material

The supplementary material for this article can be found at https://doi.org/10.1017/cts.2025.10198.
