# Supplementary material for: Evidence-based strategies for delivering grant writing skills to clinical and translational science faculty in the Mountain West
Source: J Clin Transl Sci. 2025 Nov 12;9(1):e268. doi: 10.1017/cts.2025.10198 (PMC12779486; doi:10.1017/cts.2025.10198)
Supplement: Dagda et al. supplementary material 1 — Dagda et al. supplementary material [file S2059866125101982sup001.pdf]

2022 VIRTUAL GRANT WRITING WORKSHOP

Theme: "Writing and Preparing Competitive Clinical & Translational Research Grants"

DAY ONE	DAY TWO
**DATE:** April 13th, 2022<br><br>**TIME:** 1:00-4:30 pm, Pacific Time<br>2:00-5:30 pm, Mountain Time<br><br>**LOCATION:** https://unr.zoom.us/j/82068892319	**DATE:** April 14th, 2022<br><br>**TIME:** 1:00-4:30 pm, Pacific Time<br>2:00-5:30 pm, Mountain Time<br><br>**LOCATION:** https://unr.zoom.us/j/82068892319

OBJECTIVES:

1. Recruit current & potential Principal Investigators (PIs, current and unsuccessful Pilot Grant Awardees) from the Mountain West CTR-IN affiliated Universities for grants on clinical translational research, with a focus in health disparities and community engagement research.
2. Inform the PIs on the NIH updates on grant applications and new FOAs.

SPEAKERS & CONSULTANTS:

Ruben Dagda, PhD
Associate Director, Professional Development (PD) Core, MW CTR-IN Program
Chair, Grant Writing Workshops (GWW), PD Core, MW CTR-IN Program
Chair, Advance to Funding (ATF) Program, PD Core, MW CTR-IN Program
Associate Professor, Department of Pharmacology, University of Nevada Reno

Larissa Myaskovsky, PhD
Director, PD Core, MW CTR-IN Program
Professor, University of New Mexico - School of Medicine (SOM)
Director, Ambassador Translational Research in Progress (ATRIP) Program, PD Core, MW CTR-IN Program
Director, Center for Healthcare Equity in Kidney Disease (CHEK-D)

Juli Petereit, PhD
Associate Director, Biostatistics, Epidemiology, Research & Design (BERD) Core, MW CTR-IN Program
Director, Nevada Bioinformatics Center
Co-Director Data Science Core, NIH IDeA NV INBRE

Akshay Sood, MD, PhD
Associate Director - Mentoring Unit, PD Core, MW CTR-IN Program
Professor and Miners' Colfax Medical Center Endowed Chair in Mining-Related Lung Diseases
Medical Director, Miners' Wellness Tele-ECHO Clinic
Assistant Dean, Mentoring & Faculty Retention, UNM SOM Office of Faculty Affairs & Career Development

Francisco S. Sy, MD, DrPH
Principal Investigator, MW CTR-IN Program
Professor and Chair, Department of Environmental & Occupational Health, University of Nevada, Las Vegas

2022 VIRTUAL GRANT WRITING WORKSHOP AGENDA

APRIL 13th-14th, 2022 [7 HOURS TOTAL / PACIFIC TIME]

"General Elements of the NIH Proposal"

DAY ONE – April 13th, 2022

I.	**Brief Introduction:** *FOAs in Health Disparities* - Ruben Dagda, PhD	**1:00-1:15 PM** *(15 mins.)*
II.	**Overview of the Grant Writing Process**: *The Do's and Don'ts of Writing Grants* - Ruben Dagda, PhD	**1:15-1:45 PM** *(30 mins.)*
III.	**Biostatistical Considerations to Address Rigor / Reproducibility** - Juli Petereit, PhD	**1:45-2:15 PM** *(30 mins.)*
IV.	**Writing a Competitive Specific Aims Page:** *Important Elements* - Akshay Sood, MD, PhD	**2:15-2:45 PM** *(30 mins.)*
	**BREAKOUT SESSION ONE:** Ruben Dagda, PhD & Akshay Sood, MD, PhD<br>***Workshopping the Specific Aims (SA) Page + Identify Strengths & Weaknesses in the SA Page***	**2:45-3:30 PM** *(45 mins.)*

- Participants are expected to:
  1. Present their Specific Aims (SA)
  2. Get feedback from the PD Core Team in an interactive and productive discussion

	Evaluation (Identify Strengths and Weaknesses in the SA page)	**3:30-3:45 PM** *(15 mins.)*
V.	**Significance and Innovation -** Larissa Myaskovsky, PhD	**3:45-4:25 PM** *(40 mins.)*

DAY TWO – April 14th, 2022

I.	**Developing a Competitive NIH Bio-Sketch and Current G Forms** - Larissa Myaskovsky, PhD	**1:00-1:30 PM** *(30 mins.)*
	**BREAKOUT SESSION TWO:** - Larissa Myaskovsky, PhD<br>***Identifying Strengths and Weaknesses in the Biosketch***	**1:30-2:10 PM** *(40 mins.)*
II.	**Writing a Strong Research Environment** - Larissa Myaskovsky, PhD	**2:10-2:30 PM** *(20 mins.)*
	**Break (10 minutes)**	
	**BREAKOUT SESSION THREE:** - Larissa Myaskovsky, PhD<br>***Identifying Strengths & Weaknesses in the Research Environment***	**2:40-3:20 PM** *(40 mins.)*
III.	**General Considerations Topic (K Awards)** - Larissa Myaskovsky , PhD	**3:20-3:50 PM** *(30 mins.)*
IV.	**Understanding NIH Grant & Review Process** - Francisco S. Sy, MD, DrPH	**3:50-4:20 PM** *(40 mins.)*
V.	**Talking to your PO / targeting your grant to NIH study sections:** *Tools and Tips* - Ruben Dagda, PhD	**4:20-4:50 PM** *(30 mins.)*
VI.	**Final Remarks and Follow-Up Sessions** - Ruben Dagda, PhD	**4:50-5:00 PM** *(15 mins.)*

POST EVALUATIONS SUBMITTED BY THE END OF THE WEEK

MW CTR-IN

ALASKA HAWAII IDAHO MONTANA NEVADA NEW MEXICO WYOMING

Mountain West Clinical & translational Research-Infrastructure Network Program

VIRTUAL GRANT WRITING WORKSHOP 2021

Theme: Transitioning from your pilot grant to extramural funding in health disparities and clinical translational research

DAY ONE	DAY TWO
**DATE: June 28, 2021**<br>**TIME: 1:00-5:00 pm Mountain Time**<br>**12:00-4:00 Pacific Time**<br>**LOCATION: Zoom**	**DATE: June 29, 2021**<br>**TIME: 1:00 – 4:15 pm Mountain Time**<br>**12:00-3:15 Pacific Time**<br>**LOCATION: Zoom**

OBJECTIVES:

1. Recruit current & potential Principal Investigators (PIs) from the Mountain West CTR-IN affiliated Universities for grants on clinical translational research, with an emphasis in health disparities.
2. Inform the PIs on the NIH updates on grant applications.

SPEAKERS & CONSULTANTS:

Hyeong Jun Ahn, PhD
Assist. Professor (Specialist), Univ. of Hawaii – John A. Burns SOM, Department of Quantitative Health Sciences

Bryce Chackerian, PhD
Jeffrey Michael Gorvetzian Endowed Professor of Biomedical Research, University of New Mexico
Vice Chair, University of New Mexico – School of Medicine – Dept. of Molecular Genetics & Microbiology

Ruben Dagda, PhD (Chair, Grant Writing Workshop)
Associate Professor, Department of Pharmacology, University of Nevada Reno
Associate Director, GWW and Advance to Funding Program Unit, PD Core, MW CTR-IN Program

Merle Kataoka-Yahiro, DrPH, MS, APRN
Professor, University of Hawaii at Manoa
Associate Director, Education Unit, PD Core, MW CTR-IN Program

Larissa Myaskovsky, PhD, FAST
Professor, University of New Mexico - School of Medicine (SOM)
Director, Center for Healthcare Equity in Kidney Disease (CHEK-D)
Director, PD Core, MW CTR-IN Program

Curtis Noonan, PhD
Professor of Epidemiology, College of Health Professions and Biomedical Sciences, University of Montana
Director, Pilot Projects Program (CP3) Core, MW CTR-IN Program
Director, Center for Population Health Research (CPHR)

Akshay Sood, MD, PhD
Professor and Miners' Colfax Medical Center Endowed Chair in Mining-Related Lung Diseases
Medical Director, Miners' Wellness Tele-ECHO Clinic
Assistant Dean, Mentoring & Faculty Retention, UNM SOM Office of Faculty Affairs & Career Development
Associate Director, Mentoring Unit, PD Core, MW CTR-IN Program

Grant Writing Workshop – 2021

DAY ONE AGENDA (June 28, 2021)

"General Elements of the NIH Proposal"

I. Introduction – FOAs in Health Disparities -- Chair, Ruben Dagda, PhD	***1:00 – 1:30 pm***
II. Specific Aims – Akshay Sood, MD , PhD	***1:30 – 2:00 pm***
III. Significance & Innovation Sections – Ruben Dagda, PhD & Larissa Myaskovsky, PhD	***2:00 – 2:30 pm***
IV. Research Strategy & Approach / Preliminary Data – Ruben Dagda, PhD	***2:30 – 3:00 pm***
BREAK	***3:00 – 3:15 pm***
V. Developing a Competitive NIH Bio-Sketch and Current E Forms -- Larissa Myaskovsky, PhD	***3:15 – 3:45 pm***
VI. Targeting your Grant to a Specific NIH Study Section and How to Approach the Program Official Section – Ruben Dagda, PhD	***3:45 – 4:15 pm***
VII. The NIH Review Process – Curtis Noonan, PhD	***4:15 – 4:45 pm***
VIII. Dealing with Rejection – Bryce Chackerian, PhD	***4:45 – 5:15 pm***
END OF GWW - DAY ONE	***5:15 pm***

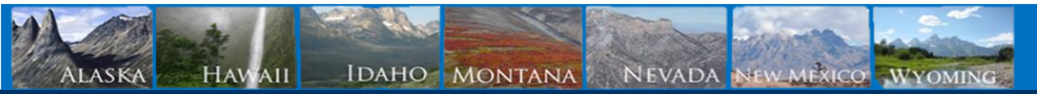

Grant Writing Workshop – 2021

DAY TWO AGENDA (June 29, 2021)

"Interactive Sessions & Specific Elements of a Health Disparities Grant Proposal"

I. Zoom Meeting Rooms: Workshopping the Specific Aims Page -- Chair, Ruben Dagda, PhD & Akshay Sood, MD, PhD	***1:00 – 1:45 pm***
II. Conducting Appropriate Power Analysis and Rigor / Reproducibility – Hyeong Jun Ahn, PhD	***1:45 – 2:15 pm***
VII. New Methods and Instruments Used in Health Disparities Research – Merle Kataoka-Yahiro, DrPH, MS, APRN	***2:15 – 3:00 pm***
BREAK	***3:00 – 3:15 pm***
VIII. Zoom Meeting Rooms: Workshopping the Research Approach and Alternative Plans, Pitfalls, Outcomes -- Chair, Ruben Dagda, PhD & PD Core Members	***3:15 – 4:15 pm***
FINAL REMARKS -- Chair, Ruben Dagda, PhD & END OF GWW - DAY TWO	***4:15 pm***

2022 GRANT WRITING WORKSHOP

*Theme: "***Preparing and Revising Competitive Clinical & Translational Research Grants***"*

DATE: Wednesday, November 16th, 2022
TIME: 10:30 am - 5:00 pm, Pacific Time
LOCATION: In-Person / JW Marriott Las Vegas Resort & Spa, Las Vegas, NV
*(**CATALUNA ROOM**) – Please look for the* **GWW** *in the conference center map*

OBJECTIVES:

1. Prepare current & potential Principal Investigators (PIs, current and unsuccessful Pilot Grant Awardees) from Mountain West CTR-IN affiliated universities to write grants focused on clinical translational research, or community engagement research.

2. Inform the PIs on NIH updates on grant applications and new FOAs.

SPEAKERS & CONSULTANTS:

Ruben Dagda, PhD
Associate Director, Professional Development (PD) Core, MW CTR-IN
Chair, Grant Writing Workshops (GWW), PD Core, MW CTR-IN
Chair, Advance to Funding (ATF) Program, PD Core, MW CTR-IN
Associate Professor, Department of Pharmacology, University of Nevada Reno

Larissa Myaskovsky, PhD
Director, Professional Development (PD) Core, MW CTR-IN
Professor, University of New Mexico, School of Medicine
Director, Ambassador Translational Research in Progress (ATRIP) Program, MW CTR-IN
Director, Center for Healthcare Equity in Kidney Disease (CHEK-D), UNM HSC

Lauren Lessard, PhD, MPH
Core Faculty, Biostatistics and Epidemiology Research Development (BERD) Core, MW CTR-IN
Associate Professor, Institute for Circumpolar Health Studies
University of Alaska, Anchorage

Juli Petereit, PhD
Co-Director, Biostatistics, Epidemiology, & Research Design (BERD) Core, MW CTR-IN
Director of Bioinformatics, University of Nevada, Reno

Akshay Sood, MD, MPH
Associate Director - Mentoring Unit, PD Core, MW CTR-IN
Professor and Miners' Colfax Medical Center Endowed Chair in Mining-Related Lung Diseases
Medical Director, Miners' Wellness Tele-ECHO Clinic
Assistant Dean, Mentoring & Faculty Retention, UNM SOM Office of Faculty Affairs & Career Development

Tony Ward, PhD
Director, Community Engagement & Outreach (CEO) Core, MW CTR-IN
Professor and Chair, University of Montana, School of Public and Community Health Sciences

2022 GRANT WRITING WORKSHOP AGENDA

I.	**Introductory Remarks and Overview of the Grant Writing Process**<br>**The Do's and Don'ts of Writing Grants**<br>Ruben Dagda, PhD	**10:30 - 11:00 am**<br>*(30 mins.)*
II.	**Writing a Competitive Specific Aims Page + Important Elements**<br>Akshay Sood, MD, MPH	**11:00 - 11:40 am**<br>*(40 mins.)*
III.	**BREAKOUT SESSION ONE:** Ruben Dagda, PhD & Akshay Sood, MD, PhD<br>***Workshopping the Specific Aims (SA) Page + Identify Strengths & Weaknesses in the SA Page***<br>❖ Participants are expected to break out in groups of three- four:<br>1. Present their Specific Aims (SA)<br>2. Get feedback from the PD Core Team in an interactive discussion	**11:40 - 12:30 pm**<br>*(50 mins.)*
IV.	***Lunch Break -*** *Follow up and discussion on identifying strengths and weaknesses on the SA page*	**12:30 - 1:00 pm**<br>*(30 mins.)*
V.	**Significance and Innovation**<br>Larissa Myaskovsky, PhD	**1:00 - 1:40 pm**<br>*(40 mins.)*
VI.	**Conducting appropriate biostatistical analysis of your preliminary data and/or developing an appropriate analysis plan for your application (Reproducibility/ Data Management Plan)**<br>Lauren Lessard, PhD, MPH	**1:40 - 2:30 pm**<br>*(50 mins.)*
VII.	**How to write an organized & compelling Research Approach Section and Preliminary Figures**<br>Ruben K. Dagda, PhD	**2:30 - 3:10 pm**<br>*(40 mins.)*
VIII.	**BREAKOUT SESSION TWO:** Ruben Dagda, PhD & Larissa Myaskovsky, PhD<br>***Workshopping the Research Approach, Data Analysis Plan, and Figures***<br>❖ Participants are expected to break out in groups of three - four:<br>1. Present their Research Approach Outline and preliminary figures<br>2. Get feedback from the PD and BERD Core Team in an interactive discussion	**3:20 - 4:00 pm**<br>*(40 mins.)*
IX.	**Talking to your Program Official & Identifying the Appropriate Study Sections for your Proposal**<br>Ruben K. Dagda, PhD	**4:00 - 4:20 pm**<br>*(20 mins.)*
X.	**How to Successfully Engage the Community**<br>Tony Ward, PhD	**4:20 - 4:50 pm**<br>*(30 mins.)*
XI.	**Closing Remarks, Plans and Follow up (Post-evaluation)**	**4:50 - 5:00 pm**<br>*(10 mins.)*
