# Supplementary material for: Evidence-based strategies for delivering grant writing skills to clinical and translational science faculty in the Mountain West
Source: J Clin Transl Sci. 2025 Nov 12;9(1):e268. doi: 10.1017/cts.2025.10198 (PMC12779486; doi:10.1017/cts.2025.10198)
Supplement: Dagda et al. supplementary material 2 — Dagda et al. supplementary material [file S2059866125101982sup002.pdf]

(Dagda et al., 2024, JCTS)

Supplementary Table 1: Written feedback from GWW participants

Virtual GWW (2022)	Positive Feedback	“I liked using breakout rooms and reviewing other's papers. Interactions between presenters and audience. The presenting team's sense of care and attentiveness toward wanting the audience to learn and grow more effectively. I liked having it virtually. My hope is I could have another shot at the training. If I could be prompted in an in-your-face way on products to send ahead of time, I could do my part to get more out of the training. Thanks, Team!
	Negative Feedback	Nothing the team could do, but the timing was terrible for me. I had a major grant due that had a very short turnaround time. It distracted me from my full participation. I do not recall getting prompts to submit examples far enough ahead of the training. Although I do not think I would have much time to give to them, I do not remember getting cues to push different products on time
In person GWW (2024)	Positive Feedback	Individualized and targeted feedback from leadership and peers. 2) Guidance on the identification of appropriate SRGs. Thank you for demystifying this process! This is an important part of developing your proposal, but it is never covered in workshops. The specific suggestions and tools provided were very helpful. Again, thank you so much for covering this! 3) Breakdown/anatomy of the specific aims page. Reiterating this may be the only page many reviewers will ever read. Suggestion to include a figure to clarify your model
	Negative Feedback	I wish that I could fully utilize the break session and hand-on activities, especially during the lunch/work session. Also, it will be appreciated if the learners are able to receive the outline of the PowerPoint presentations prior to the workshop (although I really appreciate all instructors work hard to prepare for this workshop and reviewed all assignments).
